# Broad spectrum insect resistance and metabolites in close relatives of the cultivated tomato

**DOI:** 10.1007/s10681-018-2124-4

**Published:** 2018-02-06

**Authors:** Ben Vosman, Wendy P. C. van’t Westende, Betty Henken, Henriëtte D. L. M. van Eekelen, Ric C. H. de Vos, Roeland E. Voorrips

**Affiliations:** 1 Plant Breeding, Wageningen University and Research, P.O. Box 386, 6700 AJ Wageningen, The Netherlands; 2 Bioscience, Wageningen University and Research, P.O. Box 16, 6700 AA Wageningen, The Netherlands

**Keywords:** Lycopersicon group, Whitefly, Aphid, Thrips, Caterpillar, Metabolomics, Acyl sugars, Flavonoids

## Abstract

Wild relatives of tomato possess effective means to deal with several pests, among which are a variety of insects. Here we studied the presence of resistance components against *Trialeurodes vaporariorum, Myzus persicae, Frankliniella occidentalis,* and *Spodoptera exigua* in the Lycopersicon group of *Solanum* section *Lycopersicon* by means of bioassays and comprehensive metabolite profiling. Broad spectrum resistance was found in *Solanum galapagense* and a few accessions of *S. pimpinellifolium*. Resistance to the sap sucking insects may be based on the same mechanism, but different from the caterpillar resistance. Large and highly significant differences in the leaf metabolomes were found between *S. galapagense,* containing type IV trichomes, and its closest relative *S. cheesmaniae,* which lacks type IV trichomes. The most evident differences were the relatively high levels of different methylated forms of the flavonoid myricetin and many acyl sucrose structures in *S. galapagense*. Possible candidate genes regulating the production of these compounds were identified in the *Wf*-*1* QTL region of *S. galapagense*, which was previously shown to confer resistance to the whitefly *B. tabaci.* The broad spectrum insect resistance identified in *S. galapagense* will be very useful to increase resistance in cultivated tomato.

**Electronic supplementary material:**

The online version of this article (10.1007/s10681-018-2124-4) contains supplementary material, which is available to authorized users.

## Introduction

Plants have evolved all kinds of adaptations to survive insect attacks. Their defense mechanisms interfere with the physiology and/or behaviour of the insect and may include among others the presence of (glandular) trichomes, wax layers, defensive metabolites or proteins. They are coined together as direct defense when resistance components of the plant directly interact with the attacker. As plants are facing not just one but a whole suite of attackers, they evolved a variety of mechanisms to deal with insects. Resistance mechanisms may be highly attacker-specific and interact with only one biotype of an insect, or they may be more general and protect the plant from a range of attackers (Agrawal 2011; Broekgaarden et al. 2011; Smith and Boyko 2007; Thompson 2005).

Cultivated tomato (*Solanum lycopersicum*) suffers from a wide range of arthropods, mainly generalists representing different feeding guilds. They cause direct damage through feeding, but often the greatest harm is done by the viruses they transmit. Resistance against insects and mites is found in several wild relatives of the cultivated tomato. Well known sources are *Solanum pennellii* and *S. habrochaites* (Muigai et al. 2003). Recently also some more closely related wild relatives of the cultivated tomato have been added to the list, including *S. pimpinellifolium* and *S. galapagense* showing resistance to the whitefly *Bemisia tabaci* (Firdaus et al. 2012; Lucatti et al. 2013; Rodriguez-Lopez et al. 2011), and the two-spotted spider mite, *Tetranychus urticae* (Alba et al. 2009; Rakha et al. 2017a). Currently it is unknown if also other insect species are affected by the resistance present in the Lycopersicon group of *Solanum* section *Lycopersicon* and if such resistance in the different members is governed by the same mechanism.

*Solanum pimpinellifolium* and *S. galapagense* are part of the Lycopersicon group of *Solanum* section *Lycopersicon* that also includes *S. cheesmaniae* and the cultivated tomato (Peralta and Spooner 2005; Peralta et al. 2008). Especially *S. cheesmaniae* and *S. galapagense* are genetically very close (Lucatti et al. 2013) and both species are endemic to the Galapagos islands. However, they are easily distinguishable based on leaf morphology (Darwin et al. 2003). They also differ strongly in the presence of type IV trichomes, which are present on *S. galapagense* and absent on *S. cheesmaniae* leaves (Lucatti et al. 2013). Type IV trichomes are also found on some accessions of *S. pimpinellifolium,* but not on *S. lycopersicum* (Firdaus et al. 2012). The presence/absence of type IV trichomes has been genetically mapped in an F2 population derived from a *S. lycopersicum* × *S. galapagense* cross and a major Quantitative Trait Locus (QTL) *Wf*-*1* was identified on the end of chromosome 2 (Firdaus et al. 2013).

In *Solanum* section *Lycopersicon* insect and mite resistance has been suggested to be based on specific plant compounds present in the glandular trichomes, including acyl sugars, terpenes, methyl ketones and flavonoids (Glas et al. 2012). Acyl sugars have received a lot of attention and several genes involved in the production and modification of acyl sugars have been identified (Fan et al. 2016; Schilmiller et al. 2012, 2015, 2016). Acyl sugars may be directly toxic to the insects (Luu et al. 2017), but it is also possible that their sticky nature causes death of the insect by immobilizing them on the leaf (Schilmiller et al. 2008; Rakha et al. 2017a). There are many different acyl sugars produced in a tomato plant and its wild relatives (Lucatti et al. 2013; McDowell et al. 2011), possibly exerting different effects on different insect species. However, apart from acyl sugars other compounds present in the glandular trichomes may be key to insect resistance as well. Firdaus et al. (2013) analyzed the acyl sugar content in bulks of the 10 most resistant and 10 most susceptible plants of the F2 population derived from the *S. lycopersicum* × *S. galapagense* cross and found large differences in the abundance (> 100 × difference); high abundance in resistant plants, low abundance in susceptible plants. This strongly suggests that the *Wf*-*1* QTL is also involved in acyl sugar production, although other QTLs may play a role as well.

In the current study we aimed to obtain basic information on the extent of insect resistance identified in the Lycopersicon group. More specifically we investigated (1) whether there is, next to *B. tabaci* resistance, also resistance against the phloem-feeding *Trialeurodes vaporariorum* and *Myzus persicae,* the cell content-feeding thrips *Frankliniella occidentalis,* and to the leaf feeding *c*aterpillar *Spodoptera exigua,* (2) whether the different accessions are equally effective against the different insects, and (3) which metabolites present in the leaves, as detected by using comprehensive Liquid Chromatography Mass Spectrometry (LCMS) metabolite profiling, of the different members of the Lycopersicon group correlate to resistance characteristics.

## Materials and methods

### Plant materials and growing conditions

Based on genetic relationship, resistance to *B. tabaci,* presence/absence of trichome type IV and acyl sugar profiles which we had available for approx. 100 accessions of the Lycopersicon group of *Solanum* section *Lycopersicon*, we selected 5 accessions of *S. pimpinellifolium,* 3 of *S. cheesmaniae* and 3 of *S. galapagense* for the current study to cover the variation within the group and complemented this with 4 reference accessions; see Supplementary Table 1 for details on the materials. The seeds were sown and 2 weeks later the young plants were transferred to 17 cm pots with potting compost and grown in a greenhouse at 23/19 °C (day/night temp), relative humidity (RH) 70%, light 16/8 h (day/night) at Unifarm, Wageningen, The Netherlands. Plants were randomized in blocks with one plant per block. The insect resistance evaluations started when the plants were approx. Six weeks old. Five plants per accession were used in the tests for whitefly, thrips and caterpillar resistance tests, and 7 plants per accession for the aphids resistance test.

### Insect rearings and resistance evaluations

A rearing of the whitefly *Trialeurodes vaporariorum* was obtained from Wageningen University and Research Greenhouse Horticulture, Bleiswijk, NL and maintained on tomato cv. Moneymaker. Synchronized 1 day old adult whiteflies were used for the experiment. Each plant received 3 clip-on cages containing 5 female whiteflies. The clip-on cages were placed on the abaxial side of the first, second and occasionally the third fully expanded leaves, in which numbering starts from the top of the plant, the first fully expanded leaf being the youngest. After 5 days the number of living and dead whiteflies and the number of eggs were counted.

The thrips *Frankliniella occidentalis* was obtained from the same origin and reared on 2 different hosts [common bean (*Phaseolus vulgaris*) and tomato cv. Moneymaker]. Small leaflets (5 pieces) from the first or second fully expanded leaf of each plant were inserted with their petiole in a drop 1.5% water agar that was present in a Petri dish (Falcon Tight-Fit Lid Dish 50 × 9 mm style) to prevent drying out. Five adult female thrips of assorted age were placed on the leaflet and survival of the thrips was observed at day 2, 3 and 4 after the start of the infestation. There were no significant differences observed between thrips reared on common bean versus those reared on tomato, based on the analysis of 3 plant that received bean-reared thrips and 2 plants that received tomato-reared thrips per accession (Anova thrips origin: p = 0.974) .

Eggs from the caterpillar *Spodoptera exigua* were obtained from Entocare C.V. (Wageningen, NL). Larvae were reared on tomato (cv. Moneymaker) and allowed to pupate in perlite. Adult moths were fed with 10% sucrose solution and allowed to deposit eggs on paper. Eggs were put at 25 °C and after hatching the one-day old larvae were used for the experiments. From each plant a small leaflet from the first, second and third fully expanded leaves was taken and inserted with its petiole in a drop of 1.5% water agar that was present in a Petri dish or a small container to prevent drying out. Five larvae were placed on a leaflet. When a leaflet was eaten for more than 50%, a new leaflet from the same plant was added to the Petri dish/container. The experiment was carried out in a growing chamber at 25 °C and 65% RH, 16 h day/8 h night. Larval survival and weight was measured 15 days after the start of the infestation.

A rearing of the aphid *Myzus persicae* was maintained on tomato cv. Moneymaker. Synchronized one-day old nymphs were used for the experiment. Each plant obtained 2 clip-on cages containing 5 nymphs. The clip-on cages were placed on the abaxial side of the first and second fully expanded leaves. After 12 days the number of living aphids and next generation nymphs were counted.

### LCMS profiling of leaves

With this experiment we aimed to measure the “chemical environment” of the plant leaves as experienced by the insects. However, the leaf tissue that was in direct contact with the insect, i.e. under the clip cage is not representative after an infestation (due to e.g. waxes, feces, honeydew, mold and bacterial growth). The alternative to take a sample directly outside the clip cage was also difficult as especially the *S. galapagense* type accessions have very small leaves. Therefore, we choose to sample the leaf opposite of that containing the clip cage. By using these leaves, potential differences in the leaf metabolomes due to differential growth conditions and plant development were excluded as much as possible. Leaves were harvested after finishing the bioassays, to prevent possible effects on the resistance assay. However, in this case a systemic response resulting from the aphid infestation of the opposite leaf may have occurred.

Leaves were frozen in liquid nitrogen and stored at − 80 °C until use. Each sample for metabolomics analysis contained equal amounts of leaf tissue from two plants. Frozen leaves were ground into a fine powder and 100 mg fresh weight of leaf powder was weighed for metabolite extraction with 300 µl 75% MeOH containing 0.1% formic acid (FA). Five technical replicates from a pool of samples were similarly prepared and used as so-called quality control samples (QCs) to estimate overall analytical variability for each metabolite detected (De Vos et al. 2007). LC–MS was performed on a Waters Acquity HPLC coupled to a Thermo Ion Trap-Orbitrap FTMS hybrid MS system, using a Phenomenex Luna C18 (2) column and a gradient of water and acetonitrile, both acidified with 0.1% FA, as described by (Firdaus et al. 2013). The Orbitrap FTMS was operating in negative ionization mode at a mass resolution of 60,000 (FWHM) and a mass range of m/z 90-1350 D., details in (van der Hooft et al. 2011). Metalign software (Lommen 2009) was used for unbiased mass peak picking. Extracted mass peaks corresponding to acyl sugars were annotated based on the specific accurate mass of their formic acid adduct [M + HCOOH-H]^−,^ which was calculated from the elemental formula, in combination with their specific retention times (Firdaus et al. 2013), using the Search_LC option of Metalign (Lommen et al. 2011) with a maximal mass deviation of 5 ppm and a retention time window of 0.1 min. Acyl sugars were coded according to the sugar backbone (G for glucose and S for sucrose), number of acyl chains and total number of carbon atoms in these acyl chains. For instance, S3:20 refers to sucrose acylated with 3 chains with in total 20 carbon atoms. For a number of acyl sugars, additional MSMS (= MS2) and MS3 fragmentation experiments provided information on the distribution of the individual acyl chains, e.g. S3:20 (5.5.10) refers to sucrose with acyl chains of 5, 5 and 10 carbons, respectively. Subsequently, all 114,807 mass signals extracted were aligned across all samples in an untargeted manner and the resulting peak list was further processed using MSClust (Tikunov et al. 2012), in order to group redundant mass signals originating from the same molecule, including fragments, adducts and their isotopes. The resulting list with the relative abundances (based on peak heights) of each metabolite in each sample was used for statistical analyses. Selected metabolites most significantly correlating with insect resistance were manually annotated from the observed accurate masses of the molecular ion and its fragments, as well as absorbance spectra recorded by the in-line photodiode array detector using both publicly accessible and in-house LCMS metabolite databases from previous studies on tomato fruits (Moco et al. 2007) and seedlings (Roldan et al. 2014).

### Data analysis

Per plant the observations from the Petri dishes/containers or clip-on cages were combined.

*Whitefly data* Survival was expressed as (living whiteflies)/(living + dead whiteflies); the total number of whiteflies could be less than 15 as some escaped from the cages; cages with less than 4 whiteflies and plants with less than 2 remaining cages were discarded. The numbers of eggs were divided by the estimated average number of living whiteflies present, calculated as (2*living whiteflies + dead whiteflies)/2. For ANOVA analysis these data were transformed to obtain a more or less constant residual variance: survival as arcsine[sqrt(x)] and eggs as sqrt(x).

*Thrips data* Survival was expressed as (living thrips)/(living + dead thrips). For ANOVA analysis these data were transformed as arcsine[sqrt(x)].

*Caterpillar data* When a caterpillar could not be found back (probably completely dried out), it was counted as dead, as for a caterpillar it was impossible to escape from the Petri dish/container. Survival was expressed as (living caterpillars)/(total number put on the leaf) and the total weight of the surviving caterpillars was measured in mg. For ANOVA analysis survival data were transformed as arcsine[sqrt(x)] and caterpillar weight as log(x + 0.1).

*Aphid data* Survival was expressed as (living aphids)/(living + dead aphids); the total number of aphids could be less than 10 as some escaped from the cages, and cages with less than 4 aphids were discarded. The number of next generation nymphs was divided by the estimated average number of living aphids present, calculated as (2*living aphids + dead aphids)/2. For ANOVA analysis these data were transformed to obtain a more or less constant residual variance: survival as arcsine[sqrt(x)] and number of next generation nymphs as log(x + 0.1).

For all experiments ANOVA analyses were performed with 15 accessions as treatments and 5 or 7 blocks, with corresponding degrees of freedom: 14 for the accessions, 4 or 6 for the blocks and 56 or 84 for the residuals (corrected for missing values as needed). Significance of differences of accession means was tested using an LSD test (P < 0.05) on the transformed scales.

Correlations among the accession means (of the transformed data) of the different insect resistance parameters were calculated using the Pearson’s correlation coefficient.

*Metabolite data* Non-detects were randomized between 45 and 55% of the local noise calculated by the Metalign software (at a signal to noise ratio of 3), resulting in values between 400 and 500 in the data matrix. Data were log10 transformed before carrying out t-tests (in Excel), or Principal Component Analysis (PCA) and Hierarchical Cluster Analysis (HCA), both using GeneMaths XT (Applied Maths, Belgium). A false discovery rate (FDR) correction was applied to correct for multiple comparisons. The corresponding q-values were calculated according to (Benjamini and Hochberg 1995). Before PCA and HCA the log-transformed data were normalized across samples prior to analysis. Cluster analysis was carried out using Pearson correlation and Unweighted Pair Group Method with Arithmetic Mean (UPGMA) clustering.

## Results

### Evaluation of the accessions for resistance to insects

**Fig. 1:**
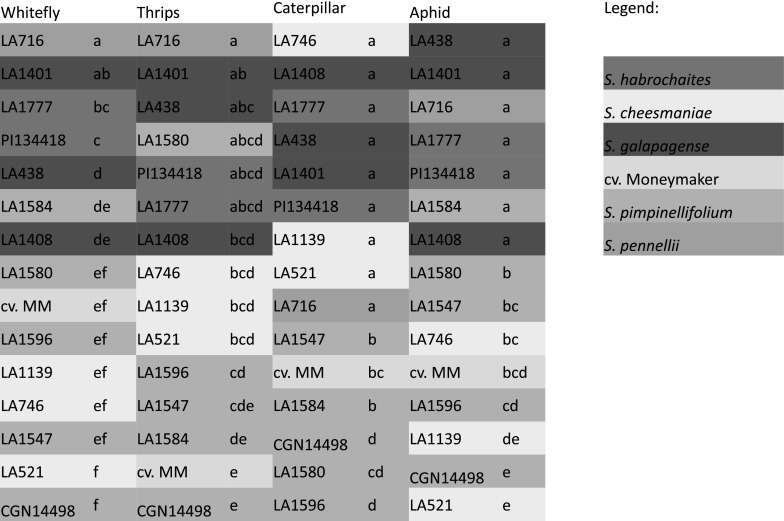
Evaluation of accessions for resistance to four insect species. Shown is the relative position of an accession in the evaluation based on the survival data for the whitefly *Trialeurodes vaporariorum,* thrips *Frankliniella occidentalis* (after 4 days), caterpillar *Spodoptera exigua* (after 4 days) and the aphid *Myzus persicae* (after 12 days). At the top of each column is the accession with the lowest survival (which was zero on the most resistant accession in each evaluation) and at the bottom the highest (which was 100% in all evaluations, except thrips). If no letters behind the accession name are in common, mean values differ significantly based on the LSD test (P < 0.05) within each evaluation. Aggregated data are based on Supplementary Tables 2–5. Different colors indicate different species. (Color figure online)

Based on available information on genetic relationships, resistance to *B. tabaci,* presence/absence of trichome type IV and acyl sugar content among 12 accessions of the Lycopersicon group of *Solanum,* section *Lycopersicon* together with 3 reference accessions (Firdaus et al. 2012) were selected for further evaluation (Supplementary Table 1) using *Trialeurodes vaporariorum, Frankliniella occidentalis, Spodoptera exigua* and *Myzus persicae.* Evaluation data of the 15 accessions are presented in a condensed form in Fig. 1, whereas the full data set can be found in the Supplementary Tables 2–5). The significance of the accession effect was < 0.001 in all experiments.

For the evaluation with the whitefly *T. vaporariorum,* two parameters (i.e. adult survival and oviposition) were used, which were highly correlated (R = 0.96, Table 1). On the *S. pennellii* accession LA716 and *S. galapagense* accession LA1401 all whiteflies died and only very few eggs were deposited (Supplementary Table 2). All *S. cheesmaniae* and most *S. pimpinellifolium* accessions were highly susceptible and almost all whiteflies survived on them.

Resistance to the thrips *F. occidentalis* was measured in a detached leaf assay where thrips survival after 2, 3 and 4 days was used as parameter. Analysis showed that differences among accessions were not significant after 2 days (ANOVA P = 0.23), but were significant after 3 (ANOVA P = 0.004) and 4 (ANOVA P < 0.001) days (Add. Table 3). Thrips survival on days 3 and 4 were highly correlated (R = 0.88, Table 1). After four days, all thrips were dead on the resistant accessions. Again the accession LA716 and LA1401 were among the most resistant, together with *S. galapagense* accession LA438, *S. pimpinellifolium* accession LA1580, and the *S. habrochaites* accessions (PI134418, LA1777) (Fig. 1).

**Table 1:**
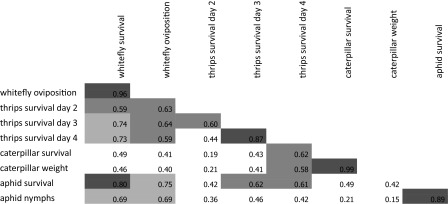
Correlation coefficients among the insect resistance parameters

Correlations were calculated using the transformed data for the different parameters for the 15 accessions. Correlations in orange, green and yellow are significantly different from 0 at P < 0.001, P < 0.01 and P < 0.05 respectively

Resistance to the caterpillar *S. exigua* was measured as larval survival and weight after 15 days in a detached leaf assay (Add. Table 4). Both parameters were highly correlated (R = 0.93, Table 1). Figure 1 shows that all *S. cheesmaniae* and *S. galapagense* accessions were among the most resistant genotypes, on which only few caterpillars survived. In contrast, all *S. pimpinellifolium* accessions and cv. Moneymaker were susceptible to *S. exigua*.

For evaluation of resistance to the aphid *M. persicae*, both survival and the number of offspring produced were used as parameters. Results are shown in Supplementary Table 5. Both parameters appeared significantly correlated (R = 0.83). The *S. galapagense* accessions were among the most resistant, together with *S. pennellii* LA716, both *S. habrochaites* accessions (LA1777, PI134418) and the *S. pimpinellifolium* accession LA1580, whereas the *S. cheesmaniae* accessions were among the most susceptible (Fig. 1).

Table 1 shows that the correlations between whitefly survival, thrips survival and aphid survival were high, whereas the correlations of these traits with caterpillar survival were low.

### Metabolite profiles

**Fig. 2:**
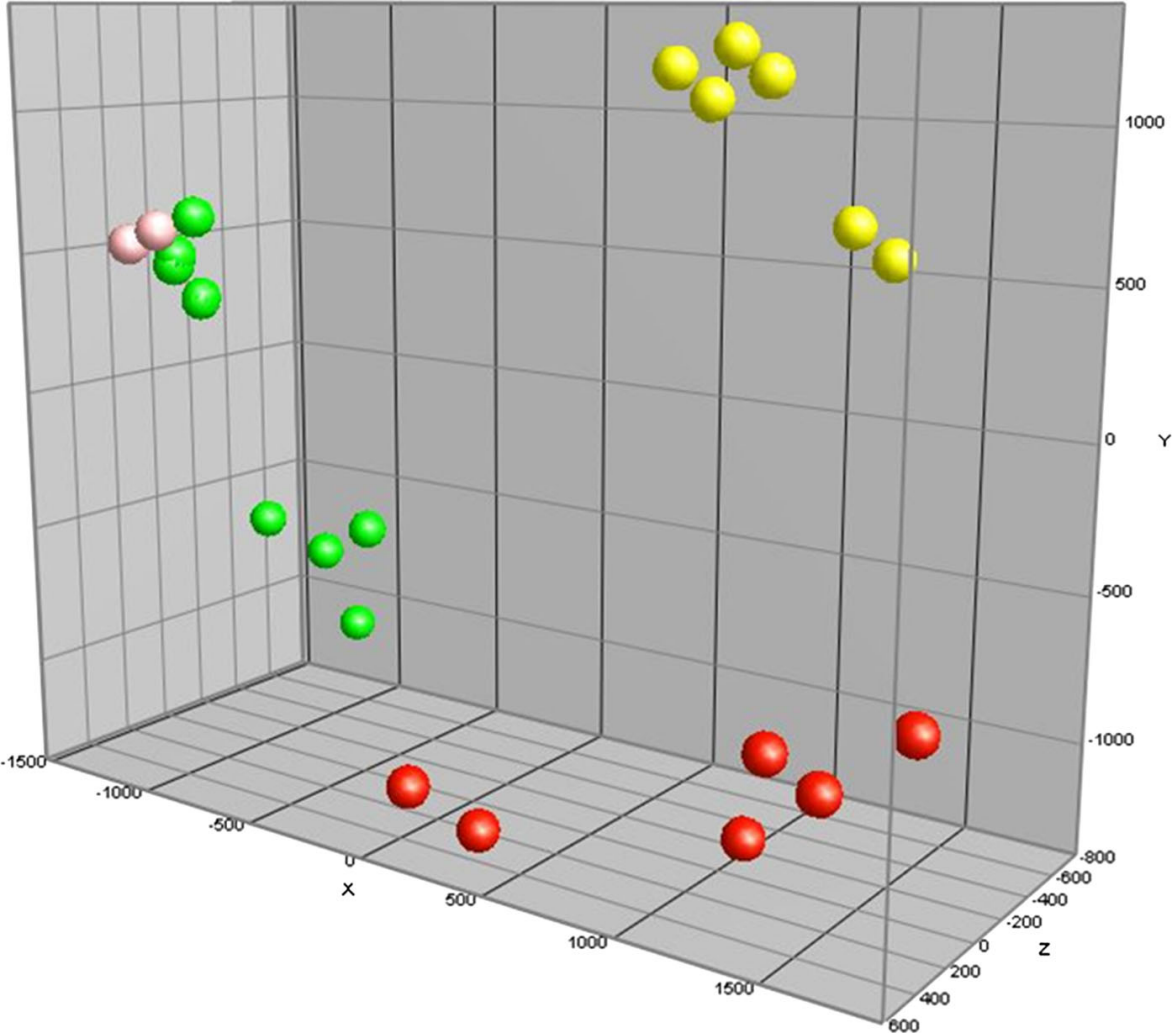
PCA analysis of the accessions based on all 2565 metabolites detected by LCMS. Accessions were analyzed in 2 independent biological replicates. Yellow spheres = *S. cheesmaniae* samples, red = *S. galapagense*, green = *S. pimpinellifolium,* and pink = cv. Moneymaker. Explained variances are 25.5, 19.7 and 8.3% for the x, y, z-axis, respectively. (Color figure online)

Differences in metabolite profiles between leaves of the Lycopersicon group accessions were analyzed using an LCMS-based, essentially untargeted metabolomics approach (De Vos et al. 2007). The extraction of leaves with 75% methanol (acidified with 0.1% formic acid), analysis of the crude extracts by high mass resolution LCMS (Supplementary Fig. 1) followed by unbiased data processing resulted in a total of 2565 putative compounds, including secondary metabolites such as alkaloids, flavonoids, phenylpropanoids, acyl sugars, etcetera, detected among the accessions evaluated (Supplementary Table 6). The PCA plot based on the metabolite profiles (Fig. 2) shows a separation of plants into 3 distinct clusters: one for the *S. galapagense* accessions, one for the *S. cheesmaniae* accessions, and one for *S. pimpinellifolium* accessions plus cv. Moneymaker. Biological replicates per accession clearly group together, thereby validating the untargeted metabolomics approach applied as a reproducible, comprehensive phytochemical phenotyping tool enabling the identification of genetic effects on the plant metabolome (see also Supplementary Fig. 1 which shows the HCA dendrogram that was constructed based on these metabolites). Within *S. pimpinellifolium* there is a division between, on the one hand, LA1580 and LA1584 and, on the other hand, LA1547, LA1596 and CGN14498 together with tomato cv Moneymaker.

**Fig. 3:**
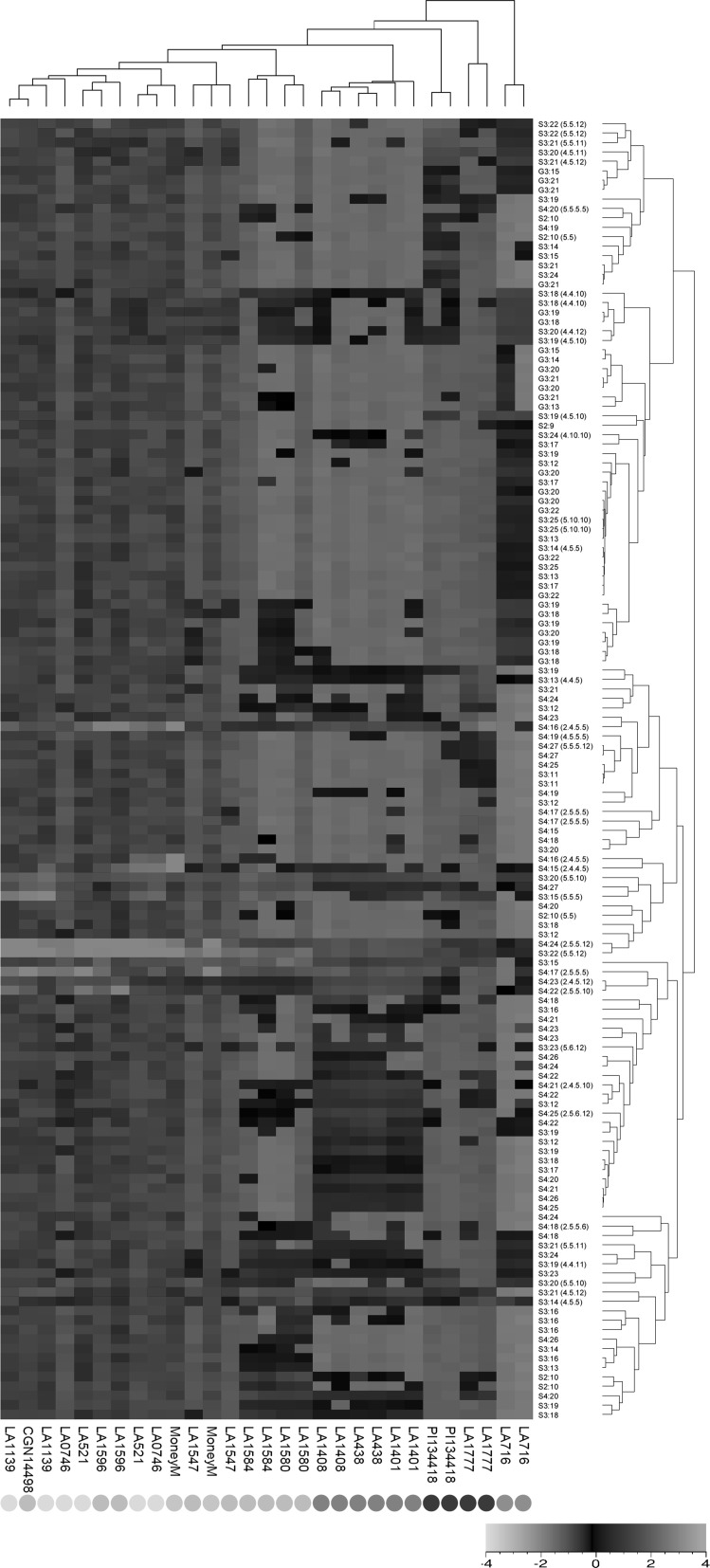
UPGMA analysis based on 138 acyl sugars detected in the 15 *Solanum* accessions. Accession number is indicated at the bottom. Species to which an accession belongs is indicated by a colored square: light brown = *S. pennellii*, dark brown = *S. habrochaites*, pink = *S. lycopersicum*, green = *S. pimpinellifolium*, yellow = *S. cheesmaniae*, red = *S. galapagense*. Dendrograms on the right side (relation among acyl sugars) and top (relation among accessions) are based on Pearson correlation coefficients. The heat map is based on all acyl sugars annotated in Supplementary Table 7). Heat map color key: red high (+ 4), green low (− 4) relative abundance of the compound. (Color figure online)

Based on their specific accurate mass and retention times in the LCMS profiles, we were able to putatively annotate 138 different acyl sugar structures of which 41 were further characterized using MSMS and MS3 fragmentation (Supplementary Fig. 2). The different tomato species appear to produce different sets of acyl sugars (Fig. 3). Unique acyl sugars, i.e. acyl sugar structures detectable in only a single accession or species, were found for all species except cv. Moneymaker and the *S. cheesmaniae* accessions. Acylated forms of glucose, i.e. acyl glucoses, were detected not only in *S. pennellii,* conform earlier reports (Schilmiller et al. 2016) but also in some lines of *S. pimpinellifolium and S. galapagense*, although most of these acyl glucoses were present at relatively low levels as compared to *S. pennellii*. Both accessions of *S. habrochaites* accumulated a unique set of acyl sucroses. Likewise, the *S. pimpinellifolium* accessions LA1580 and LA1584 produced unique acyl sucroses, among which were 2 isomers of S2:10, i.e. sucrose acylated with only 2 acyl groups with a total length of 10 carbons. *Solanum galapagense* produced several unique S3 and S4 acyl sucroses (Supplementary Table 7).

The genetically closely-related species *S. galapagense* and *S. cheesmaniae* are clearly different at their metabolite level, as shown in Supplementary Fig. 3. Between the two species 260 metabolites significantly differed in their relative abundance (FDR = 0.01), i.e. 10% of all metabolites detected. Of these differential metabolites 76 showed a *S. galapagense*/*S. cheesmaniae* (SG/SC) ratio < 0.5 and 175 a SG/SC ratio > 2. Sixty-four metabolites were at least 100 times more abundant in *S. galapagense,* while 9 were 100 times more abundant in *S. cheesmaniae* (Supplementary Table 6). Out of the 5 metabolites with the highest SG/SC ratio, 4 represented different methylated forms of myricetin, a flavonol-type of flavonoid. These metabolites were all absent, i.e. below the detection limit, in *S. cheesmaniae*. Between *S. galapagense* and *S. cheesmaniae* accessions there were 39 differentiating acyl sugars, all of them with a sucrose backbone; 23 of these were at least 100 times more abundant in *S. galapagense* than in *S. cheesmaniae* (Supplementary Table 7).

## Discussion

### Insect resistance in the Lycopersicon group of *Solanum* section *Lycopersicon*

There are large and highly significant differences in insect resistance among accessions from the Lycopersicon group. When evaluating the sap sucking insects (whitefly, aphid) and the cell content feeder (thrips), the accessions from *S. galapagense* were among the most resistant, together with the reference accessions from *S. pennellii* and *S. habrochaites* which previously have been shown to be highly resistant to insects (Kennedy 2003). Within *S. pimpinellifolium*, resistance to some pest insects is found, i.e. thrips resistance in accession LA1580 and aphid resistance in LA1584. The other accessions of *S. pimpinellifolium* were very susceptible, even more susceptible than cv. Moneymaker. Accessions of *S. cheesmaniae* are as susceptible to sap sucking insects as cv. Moneymaker. The results obtained for *S. galapagense* and *S. cheesmaniae* accessions with regard to their contrasting resistance to the whitefly *T. vaporariorum*, as shown in the present study, are very similar to those previously reported for *B. tabaci* (Lucatti et al. 2013; Rakha et al. 2017b), suggesting a similar mechanism governing resistance against both whitefly species. The same mechanism may also account for resistance against thrips and aphids, as the data for adult survival of whitefly, thrips and the aphids are highly correlated. All insect resistant accessions possess glandular trichomes type I and IV on their leaves, the vast majority of them being type IV, strongly suggesting that these trichomes are essential for plant resistance, as was shown previously (Firdaus et al. 2012; Lucatti et al. 2013). Considering the plant tissues on which whiteflies, aphids and thrips feed, it is unlikely that these insects will ingest phytochemicals present in the glandular trichomes. However, their bodies and wings may get into direct contact with compounds actively secreted by, or released from, the type I and/or IV trichomes. It has been suggested that the sticky contents released by these trichomes can immobilize the insects on the leaf surface, ultimately leading to the death of the insect (Rakha et al. 2017a; Schilmiller et al. 2008). Indeed, we observed dead insects “glued” onto the leaves of trichome type I and IV containing accessions in our clip cage assays.

Interestingly, the results obtained for the chewing *S. exigua* larvae were quite different from those obtained for the sap sucking and cell content feeding pest insects. The *S. cheesmaniae* accessions show a *S. exigua* resistance level similar to that of *S. galapagense* accessions, suggesting that resistance against *S. exigua* is likely based on a different mechanism (or compounds) than the resistance against the sap suckers. Larvae of *S. exigua* consume whole leaves and are thus ingesting and exposed to all compounds present in the various leaf cells, including the trichomes, while sap suckers are only ingesting phloem sap. All *S. pimpinellifolium* accessions were very susceptible to *S. exigua*, suggesting that the hypothetical resistance related compound(s) is absent in this species. However, many metabolites were found to be differential between the, relatively few, *S. pimpinellifolium* and *S. cheesmaniae* accessions investigated in the present study (Supplementary Tables 1 and 7). To get more insight into the key compound(s) involved in *S. exigua* resistance it is necessary to compare larger numbers of different accessions from both *S. pimpinellifolium* and *S. lycopersicum*, on the one hand, and both *S. galapagense* and *S. cheesmaniae,* on the other hand. Alternatively, segregating populations of *S. galapagense* × *S. lycopersicum* (or *S. pimpinellifolium*) crosses may be used.

### Leaf metabolites and their relation to the *Wf1*-QTL

Accessions from the three wild species *S. pimpinellifolium, S. galapagense* and *S. cheesmaniae* form distinct groups at their metabolite level. Within the *S. pimpinellifolium* accessions, there appear to be two distinct groups based on differences in their metabolite profiles: one containing the accessions LA1580 and LA1584 and the other containing LA1547, LA1596 and CGN14498. This division within *S. pimpinellifolium* accessions is related to the presence/absence of type IV trichomes, which are absent in the latter three accessions.

Despite the small differences at their genome level (Lucatti et al. 2013), large differences exist in the metabolomes between *S. galapagense* and *S. cheesmaniae* accessions. The abundances of 260 metabolites were significantly different between the two species, which is about 10% of the total number of detected metabolites. Such large differences may be due to common biochemical processes, clustering of genes involved in the synthesis of metabolites or regulatory mechanisms affecting the presence/absence of trichomes type I and IV. In a previous paper, using a *S. galapagense* × *S. lycopersicum* F2 population, we have shown that both whitefly resistance and the production of type IV trichomes is controlled by a major QTL, designated *Wf*-*1*, located on the end of Chromosome 2. In addition, resistant and susceptible plants of this F2 population showed large differences in the composition and content of acyl sugars, relate to the presence/absence of the type IV trichomes (Firdaus et al. 2013). The *S. galapagense* and *S. cheesmaniae* plants analyzed here show a similar contrast in whitefly resistance related to the presence or absence of trichome type IV and acyl sugar content. Therefore we speculate that the same *Wf*-*1* QTL, identified by the *S. galapagense* × *S. lycopersicum* crossing, is also underlying the observed resistance-related differences between *S. galapagense* and *S. cheesmaniae,* and evaluated this region for candidate genes related to the production of differentially expressed metabolites and/or the production of the glandular trichome itself. Among the compounds with the highest *S. galapagense*/*S. cheesmaniae* (SG/SC) ratios, different *O*-methylated forms of myricetin and one of quercetin, both belonging to the flavonol-type of flavonoids, were within the top 6 of differentially accumulated compounds (Supplementary Table 6). Myricetin methyl transferases have been identified on chromosome VI of *S. habrochaites*, and transcripts of these genes were shown to be present in glandular trichomes (Kim et al. 2014; Schmidt et al. 2011). In the *Wf*-*1* QTL region of the reference sequence Heinz 1706, between position SL2.50ch02: 53768859 and the end of the chromosome, there are four candidate genes annotated as caffeoyl-CoA *O*-methyltransferases (IPR002935 *O*-methyltransferase) which may be involved in the methylation of these flavonol-type of phenolic compounds (Supplementary Table 8)). We hypothesize that at least one of these *O*-methyltransferases can act as a flavonol *O*-methyl transferase leading to the accumulation of the *O*-methylated myricetin and quercetin. Although several flavonoids (Morant et al. 2003; Treutter 2005) have been implicated in resistance against pathogens and herbivores, it remains to be established whether these methylated forms of myricetin and quercetin are directly involved in whitefly resistance.

Between the different accessions carrying glandular trichomes type IV there are also large differences in the acyl sugar composition, in which each plant species accumulates a specific set of acyl sugars (Fig. 3). Several acyl glucoses are exclusively produced by *S. pennellii*, as was shown before (Blauth et al. 1998; McDowell et al. 2011). Other species mainly produce acyl sucroses. Relative high levels of specific acyl sugars are also found in both *S. habrochaites*, the *S. pimpinellifolium* with type IV trichomes and the *S. galapagense* accessions. The fact that species-specific acyl sugars are observed may be related to the presence or absence of specific acyl transferases (Fan et al. 2016) or CXE carboxylesterase (Schilmiller et al. 2016), which are involved in the production and modification of acyl sugars, respectively. For 39 different acyl sugar structures, i.e. almost 30% of the total acyl sugar structures identified, the relative abundance differed significantly between *S. galapagense* and *S. cheesmaniae* accessions with all of them being higher in *S. galapagense*. With regard to the production of these acyl sugars, the *Wf1*-QTL region contains one putative candidate gene harboring the IPR003480 domain encoding acyl transferases (Fan et al. 2016). This candidate gene is annotated as a *N*-hydroxycinnamoyl/benzoyltransferase 3, and it remains to be established whether the corresponding protein is able to esterify glucose and/or sucrose molecules with aliphatic carbon chains to produce acyl sugars. In the same *Wf1*-QTL region there is also one gene that encodes a putative CXE carboxylesterase, which may be involved in modification of acyl sugars (Schilmiller et al. 2016). It should be noted that the candidate genes mentioned above may also show up if they are closely linked to the actual causal gene.

In principle, any or all of the compounds that are significantly different between *S. galapagense* and *S. cheesmaniae* may be responsible for the differential resistance against whiteflies, aphids and thrips. For several compounds mentioned above, i.e. acyl sugars, methylated flavonols, it has been shown that these are specifically produced or accumulating in the glandular trichomes (Glas et al. 2012). The fact that a large number of metabolites is differentially expressed between *S. galapagense* and *S. cheesmaniae* suggests a common biochemical process or regulatory mechanism being responsible for the observed differences in the metabolome. Such a key process could be the formation of the type IV trichomes itself, thus regulating the production and accumulation of a large group of defense compounds. It may even be hypothesized that only the formation of the head cell is key for the accumulation of these trichome-specific metabolites. Both head cell formation and the accumulation of its specific compounds could be under control of one or more transcription factors, similar to the situation of trichome formation in *Arabidopsis thaliana* (Pattanaik et al. 2014; Zhou et al. 2014). Obviously, there will be other genes required for the production of these metabolites as well, so there may be additional QTLs at other places in the genome for several of the metabolites. The resistance controlling *Wf1*-QTL region contains several genes encoding transcription factors (see Additional Table S8). We hypothesize that at least one of these transcription factors within this *Wf1*-QTL region is involved in trichome or head cell formation, thereby regulating the accumulation of metabolites conferring insect resistance. Clearly, further research is needed to shed light on the possible involvement of candidate genes in this *Wf1*-QTL region.

### Sources of resistance that may be used to increase insect resistance in cultivated tomato

In greenhouse cultivation most pest insects can be controlled biologically by natural enemies, e.g. predators and parasitoids that are released in the greenhouse (Hoddle et al. 1998; Van Lenteren 2000). However, in the open field the effect of biological control is not sufficient to remain below the damage threshold levels, and thus insecticides are used frequently. Yet, some insect species are difficult to control with insecticides and they develop resistance to the insecticides very quickly (Alyokhin et al. 2015). In addition, several of the insecticides are hazardous to the environment, which also urges for alternative solutions to reduce or prevent damage caused by insects. The best strategy is to prevent the insect problems from occurring by using resistant varieties. Tomato wild relatives constitute a large resource for all kinds of traits, which may be used to improve cultivated tomato. However, attempts to use wild relatives as sources of resistance have until now not resulted in insect resistant tomatoes (Lawson et al. 1997; Leckie et al. 2012). This is most likely due to the complex genetics of the resistance mechanism within these species, involving several QTLs (van den Oever-van den Elsen et al. 2016). Also, crossings with wild species are often difficult and linkage drag is not easily removed by back-crossing. The recently identified sources of insect resistance in the Lycopersicon group of *Solanum* section *Lycopersicon* may solve this problem to a large extent, as species within this group are closely related to the cultivated tomato and easier to cross, and also may carry less unfavourable alleles. Based on the results presented here and by others (Rakha et al. 2017a, b), especially the *S. galapagense* accessions are very promising for future breeding efforts towards improved, insect resistant tomato varieties. The resistance in *S. galapagense* is a broad spectrum type of resistance, largely based on the presence of glandular trichomes and its phytochemical content. The presence-absence of type IV trichomes was mapped to the *Wf1*-QTL on the end of chromosome II of *S. galapagense,* which is also responsible for *B. tabaci* resistance (Firdaus et al. 2013). Therefore, it is likely that this specific QTL also governs the resistance towards the other insects. Such a broad spectrum resistance is especially useful in open field cultivation systems, where a whole suite of insects can freely fly and infest the crop.

## Conclusions

A broad spectrum resistance towards insects has been identified in close relatives of the cultivated tomato. Trichome type IV containing species from the Lycopersicon group of *Solanum* section *Lycopersicon* show resistance to a range of pest insects. Especially accessions of *S. galapagense* are highly resistant. Comparison of the metabolite content of *S. galapagense* to the genetically very similar *S. cheesmaniae*, which does not contain type IV trichomes, shows large differences in the levels of both acyl sugars and several O-methylated forms of the flavonol myricetin. The *Wf1*-QTL region previously identified by us contains several structural and transcription factor genes that may be responsible for the observed differences in insect resistance and metabolites between the two species. The broad spectrum insect resistance of *S. galapagense* holds great promises for transfer into cultivated tomato and will be especially useful in open field cultivation systems where a whole suite of insects can attack the crop.

## Electronic supplementary material

Below is the link to the electronic supplementary material.

Supplementary material Table S1: List of plant materials used in screening for insect resistance. Table S2: Evaluation data for whitefly (*Trialeurodes vaporariorum*) resistance. Table S3: Evaluation data for thrips (*Frankliniella occidentalis*) resistance. Table S4: Evaluation data for *Spodoptera exigua* resistance. Table S5: Evaluation data for aphid (*Myzus persicae*) resistance. Table S6: All metabolites detected in the LCMS analysis of the accessions studied. Table S7: Acyl sugars detected (only FA adducts) in leaves of the accessions studied. Table S8: Genes in the 2-LOD interval of the *Wf*-*1* QTL region of the Heinz genome sequence (version SL2.50) (XLSX 1727 kb)

Supplementary material Fig. 1: Contrasting LC-LTQ-Orbitrap FTMS profiles of tomato leaf extracts from *S. galapagense* LA1401 (A, C) and cv. Moneymaker (B, D). Upper traces A and B show the detector response at full scan range, while lower traces C and D show the chromatogram of m/z 737.3965 (formic acid adduct of acyl sugar S22) at a window of 5 ppm mass deviation (PDF 42 kb)

Supplementary material Fig. 2: LC-LTQ-Orbitrap FTMS characterization of acyl sugars in tomato leaves. The chromatographic peak eluting at a retention time 40.35 min, which was identified as acyl sugar S3:22 (5.5.12), is shown here as an example (PDF 104 kb)

Supplementary material Fig. 3: UPGMA analysis of the accessions used, based on the contents of 2565 metabolites. Pearson correlation was used as similarity measure. Bootstrap values are indicated with the branches. The colored bar indicates the species to which an accession belongs: green *S. pimpinellifolium*, yellow *S. cheesmaniae*, red *S. galapagense*, and pink cv. Moneymaker. Below the dendrogram is a heat map of part (49) of the 2565 metabolites that is specific to *S. galapagense.* Heat map color key: red high (+4), green low (-4) concentration of the compound (TIFF 358 kb)
