# Supplementary material for: Broad spectrum insect resistance and metabolites in close relatives of the cultivated tomato
Source: Euphytica. 2018 Feb 6;214(3):46. doi: 10.1007/s10681-018-2124-4 (PMC6445503; doi:10.1007/s10681-018-2124-4)
Supplement: Supplementary file 2 — Supplementary material Fig. 1: Contrasting LC-LTQ-Orbitrap FTMS profiles of tomato leaf extracts from S. galapagense LA1401 (A, C) and cv. Moneymaker (B, D). Upper traces A and B show the detector response at full scan range, while lower traces C and D show the chromatogram of m/z 737.3965 (formic acid adduct of acyl sugar S22) at a window of 5 ppm mass deviation (PDF 42 kb) [file 10681_2018_2124_MOESM2_ESM.pdf]

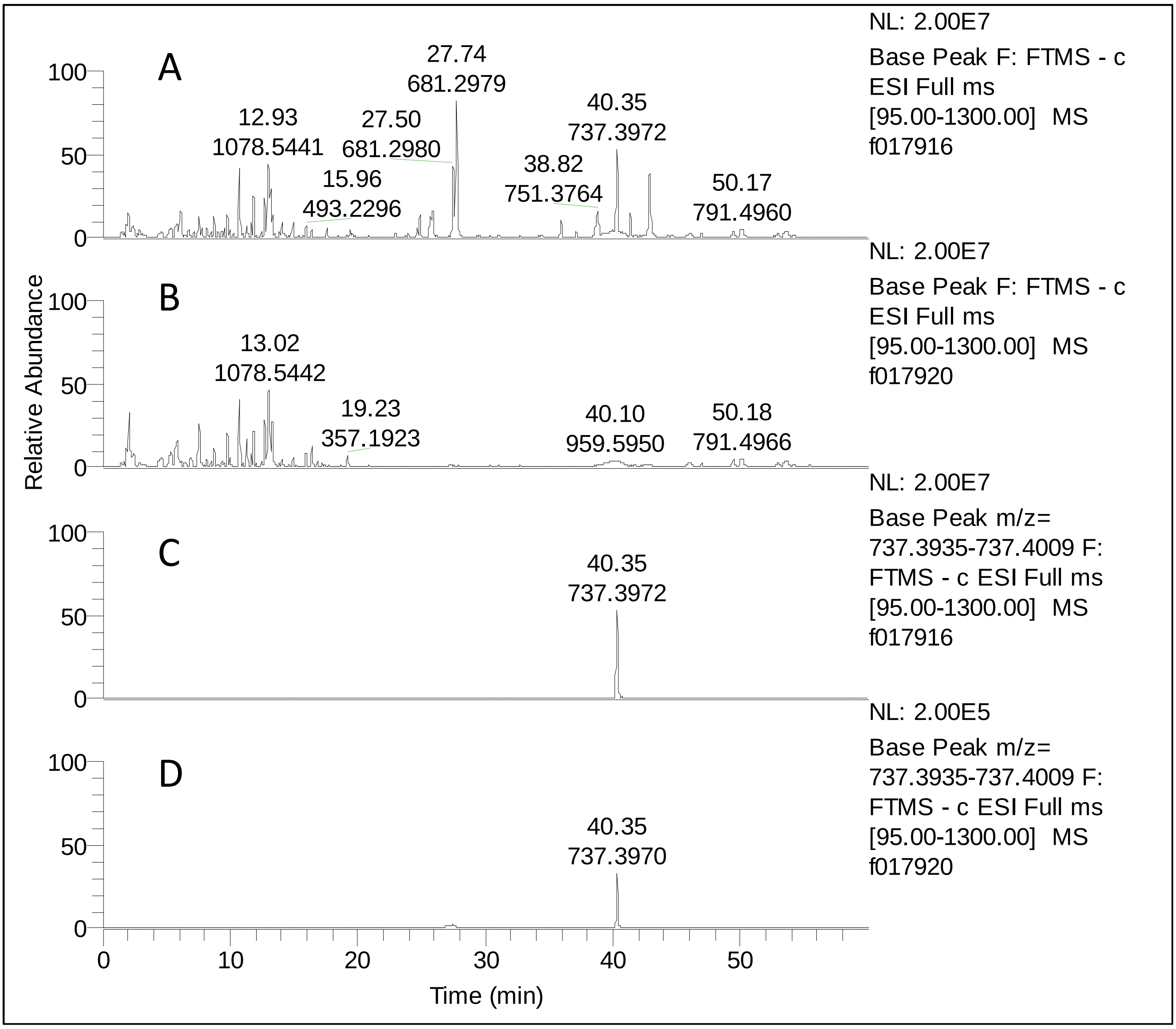

Supplemental Figure 1: Contrasting LC-LTQ-Orbitrap FTMS profiles of tomato leaf extracts from *S. galapagense* LA1401 (A, C) and *S. lycopsersicon* var. MoneyMaker (B, D). Upper traces A and B show the detector response at full scan range, while lower traces C and D show the chromatogram of m/z 737.3965 (formic acid adduct of acyl sugar S22) at a window of 5 ppm mass deviation. Values indicated above the chromatographic peaks indicate their retention time and exact mass of base peak (highest m/z signal) on top of peaks, respectively. Note that Y-scale (detector response) for traces A, B and C is set at $2x10^7$ ions per scan, while Y-scale for trace D is set at $2x10^5$ ions. Thus, the relative level of acyl sugar S22 at retention time 40.35 min is about 150 fold higher in LA1401 compared to cv. Moneymaker.
