# Supplementary material for: Broad spectrum insect resistance and metabolites in close relatives of the cultivated tomato
Source: Euphytica. 2018 Feb 6;214(3):46. doi: 10.1007/s10681-018-2124-4 (PMC6445503; doi:10.1007/s10681-018-2124-4)
Supplement: Supplementary file 3 — Supplementary material Fig. 2: LC-LTQ-Orbitrap FTMS characterization of acyl sugars in tomato leaves. The chromatographic peak eluting at a retention time 40.35 min, which was identified as acyl sugar S3:22 (5.5.12), is shown here as an example (PDF 104 kb) [file 10681_2018_2124_MOESM3_ESM.pdf]

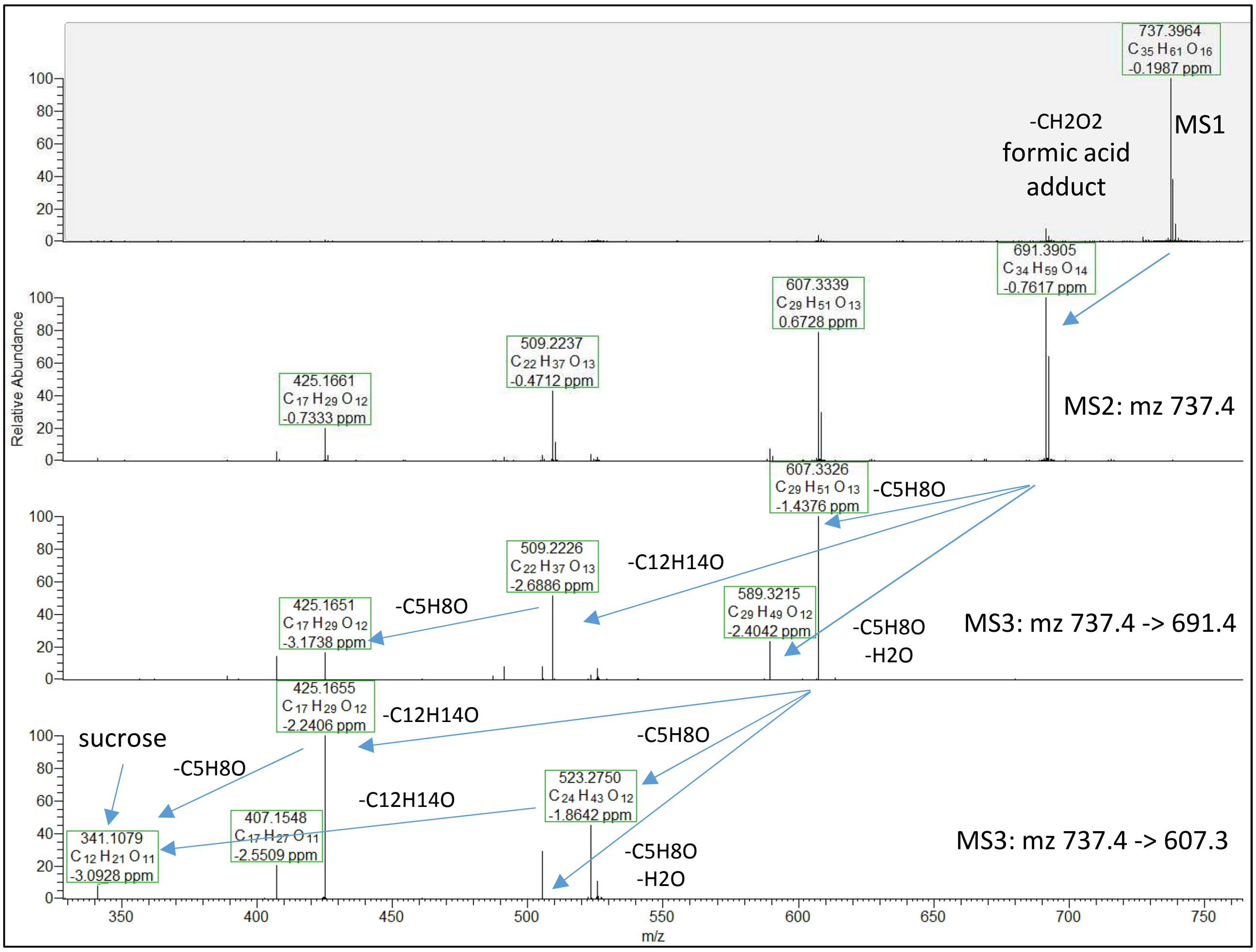


Supplemental Figure 2: LC-LTQ-Orbitrap FTMS characterization of acyl sugars in tomato leaves. The chromatographic peak eluting at a retention time 40.35 min is shown here as an example. As indicated in the green box above the mass peak, the compound was detected by the Orbitrap FTMS in full scan mode (MS1) at an exact mass of m/z 737.3964, corresponding to an elemental formula of C35H61O16 within a mass deviation of only 0.1987 ppm. This mass was subsequently subjected to sequential fragmentation in the LTQ -ion trap while eluting from the column, and its fragments were detected by the Orbitrap FTMS at high mass resolution (Van der Hooft et al 2012). In MSMS mode (MS2) this mass 737.4 lost a formic acid adduct thereby revealing the actual molecular ion, $[M-H]^-$, with an exact mass of 691.3905, i.e. C34H59O14, as the highest mass signal. Subsequent fragmentation (MS3) of this m/z 691.4 resulted in losses of both C5H8O (with or without additional H2O) and C1212O fragments, while fragmentation of the second highest MS2 signal 607.3, i.e. the molecular ion minus one C5H8O side chain, again showed both losses of C5H8O and C1214O as well as the presence of the sucrose ($[M-H]^-$) backbone. The compound detected with m/z 737.3964 was thus indicated as acyl sugar S3:22 (5.5.12) with retention time 40.35 min.
