# Supplementary figures and images for: Broad spectrum insect resistance and metabolites in close relatives of the cultivated tomato
Source: Euphytica. 2018 Feb 6;214(3):46. doi: 10.1007/s10681-018-2124-4 (PMC6445503; doi:10.1007/s10681-018-2124-4)

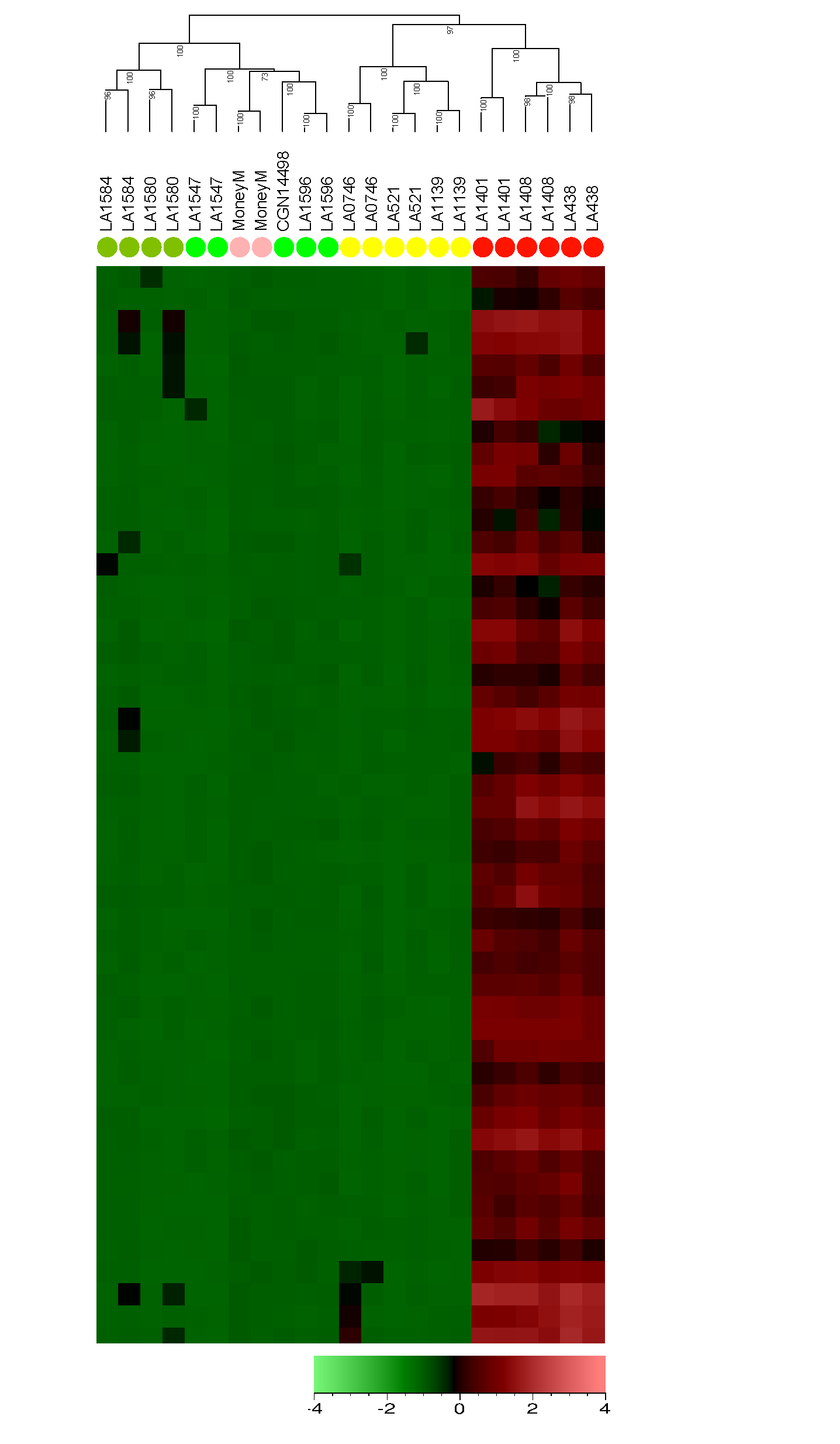

Supplement: Supplementary file 4 — Supplementary material Fig. 3: UPGMA analysis of the accessions used, based on the contents of 2565 metabolites. Pearson correlation was used as similarity measure. Bootstrap values are indicated with the branches. The colored bar indicates the species to which an accession belongs: green S. pimpinellifolium, yellow S. cheesmaniae, red S. galapagense, and pink cv. Moneymaker. Below the dendrogram is a heat map of part (49) of the 2565 metabolites that is specific to S. galapagense. Heat map color key: red high (+4), green low (-4) concentration of the compound (TIFF 358 kb) [file 10681_2018_2124_MOESM4_ESM.tif]
